# Evaluation of lesion contrast in the walk-through long axial FOV PET scanner simulated with XCAT anthropomorphic phantoms

**DOI:** 10.1186/s40658-024-00645-z

**Published:** 2024-05-09

**Authors:** Meysam Dadgar, Jens Maebe, Stefaan Vandenberghe

**Affiliations:** Department of Electronics and Information Systems, Medical Image and Signal Processing, Ghent University, C. Heymanslaan 10, Ghent, Belgium

**Keywords:** Walk-through PET, GATE simulation, Total-body PET, Lesion, XCAT phantom, Biograph Vision Quadra

## Abstract

**Background:**

This study evaluates the lesion contrast in a cost-effective long axial field of view (FOV) PET scanner, called the walk-through PET (WT-PET). The scanner consists of two flat detector panels covering the entire torso and head, scanning patients in an upright position for increased throughput. High-resolution, depth-of-interaction capable, monolithic detector technology is used to provide good spatial resolution and enable detection of smaller lesions.

**Methods:**

Monte Carlo GATE simulations are used in conjunction with XCAT anthropomorphic phantoms to evaluate lesion contrast in lung, liver and breast for various lesion diameters (10, 7 and 5 mm), activity concentration ratios (8:1, 4:1 and 2:1) and patient BMIs (18–37). Images were reconstructed iteratively with listmode maximum likelihood expectation maximization, and contrast recovery coefficients (CRCs) were obtained for the reconstructed lesions.

**Results:**

Results shows notable variations in contrast recovery coefficients (CRC) across different lesion sizes and organ locations within the XCAT phantoms. Specifically, our findings reveal that 10 mm lesions consistently exhibit higher CRC compared to 7 mm and 5 mm lesions, with increases of approximately 54% and 330%, respectively, across all investigated organs. Moreover, high contrast recovery is observed in most liver lesions regardless of diameter or activity ratio (average CRC = 42%), as well as in the 10 mm lesions in the lung. Notably, for the 10 mm lesions, the liver demonstrates 42% and 62% higher CRC compared to the lung and breast, respectively. This trend remains consistent across lesion sizes, with the liver consistently exhibiting higher CRC values compared to the lung and breast: 7 mm lesions show an increase of 96% and 41%, while 5 mm lesions exhibit approximately 294% and 302% higher CRC compared to the lung and breast, respectively.

**Conclusion:**

A comparison with a conventional pixelated LSO long axial FOV PET shows similar performance, achieved at a reduced cost for the WT-PET due to a reduction in required number of detectors.

## Background

Over the past few decades, PET/CT scanners have played a pivotal role in the detection, localization, characterization, and staging of cancer [1, 2]. However, despite significant advancements, the limited axial field-of-view (FOV) of PET scanners (ranging from 15 to 30 cm) poses a barrier to further improvements. This limitation has spurred a new area of research in the field of nuclear imaging, aiming to develop PET scanners with longer axial FOV capable of covering the entire patient body in a single bed position [3, 4]. These scanners are commonly referred to as total-body (TB) PET scanners. Notably, the uEXPLORER [3, 5], PennPET Explorer [6, 7] and Biograph Vision Quadra PET/CT [8, 9] are recognized as the world’s first TB-PET scanners. Extending the axial FOV and providing greater detector coverage, TB-PET scanners significantly enhance the detection probability of emitted photons, thereby increasing sensitivity compared to conventional PET scanners [10, 11]. Therefore, an increased signal-to-noise ratio, possibility for shorter patient scan times or reduced injection doses, and enhancement in the detection of smaller lesions are some of the main advantages of TB-PET scanners [2]. Such scanners therefore show considerable promise for improving the overall relevance of PET imaging in cancer diagnosis.

Despite the impressive performance of TB-PET scanners, their high construction price and maintenance costs have emerged as significant barriers to their widespread utilization [2, 3]. The axial length of PET scanners plays a crucial role in determining the final construction price of these scanners. Extending the axial FOV of PET scanners requires a larger quantity of electronic components, silicon photomultipliers (SiPMs), and scintillation material [12]. Consequently, a pertinent question arises regarding the optimal axial length for TB-PET scanners, considering the need to balance cost and performance.

Recognizing the crucial role of TB-PET scanners in facilitating more efficient patient diagnosis, numerous ongoing efforts are centered on their development, focusing on affordable alternative systems. These initiatives are characterized by innovative designs in detector configurations and the utilization of cost-effective scintillation materials, aiming to make these advanced diagnostic tools more accessible [12–18].

This pursuit has become a prominent and widely discussed topic among scientists in the field, driven by the urgent need to make TB-PET scanners more accessible and cost-effective while maintaining their diagnostic efficacy.

This study focuses on the performance of a new, cost-efficient, long axial FOV (LAFOV) PET design: the walk-through (WT) PET [12, 19–24]. The scanner consists of two vertically positioned flat panels containing high-resolution, depth of interaction (DOI) capable, monolithic detectors [24]. The panels, 74 cm × 106 cm in size with a 50 cm gap in between (AFOV = 106 cm), provide simultaneous head and torso imaging. The patient is scanned in upright position during an envisioned acquisition time of only 30 s.

In our preceding research, we conducted an investigation into the performance of the WT-PET scanner, setting it against a conventional LAFOV scanner with design based on the Siemens Quadra, henceforth referred to as LSO LAFOV PET.

This study encompassed an evaluation of the NEMA characteristics, with a parallel verification through experimental results obtained from the Siemens Quadra [19]. Our findings revealed the superior spatial resolution of the WT-PET scanner, which remained below 2 mm across its entire imaging volume, due to its use of DOI capable monolithic detectors. Additionally, WT-PETs sensitivity (154.0 cps/kBq) was found to be on par with the values reported by Prenosi et al. (175.4 cps/kBq) [9], underscoring the WT-PET’s efficacy and potential as a reliable imaging tool in the realm of PET technology.

Table 1 describes the configuration and system characteristics of the WT-PET as investigated in this study, alongside the LAFOV LSO PET, which was utilized to compare the performance of our proposed scanner in the discussion section of this paper [19].

**Table 1:** The system characteristics of walk-through PET and LSO LAFOV PET

Parameters	WT-PET	LSO LAFOV PET
Panel distance (cm)	50	–
Ring diameter (cm)	–	82
Panel dimensions (cm)	74 × 106	–
Scintillation material	BGO	LSO
Scintillation dimensions (mmˆ3)	16 × 50 × 50	3.2 × 3.2 × 20
Average FWHM @ (1,0,0) (mm)	1.37	2.67
Sensitivity @ center (cps/kBq)	154.0	179.7
Scatter fraction	30.72	36.18
Time window (ns)	5	4.7
TOF resolution (ps)	327	228
Energy window (KeV)	434–645	455–645

**Fig. 1:**
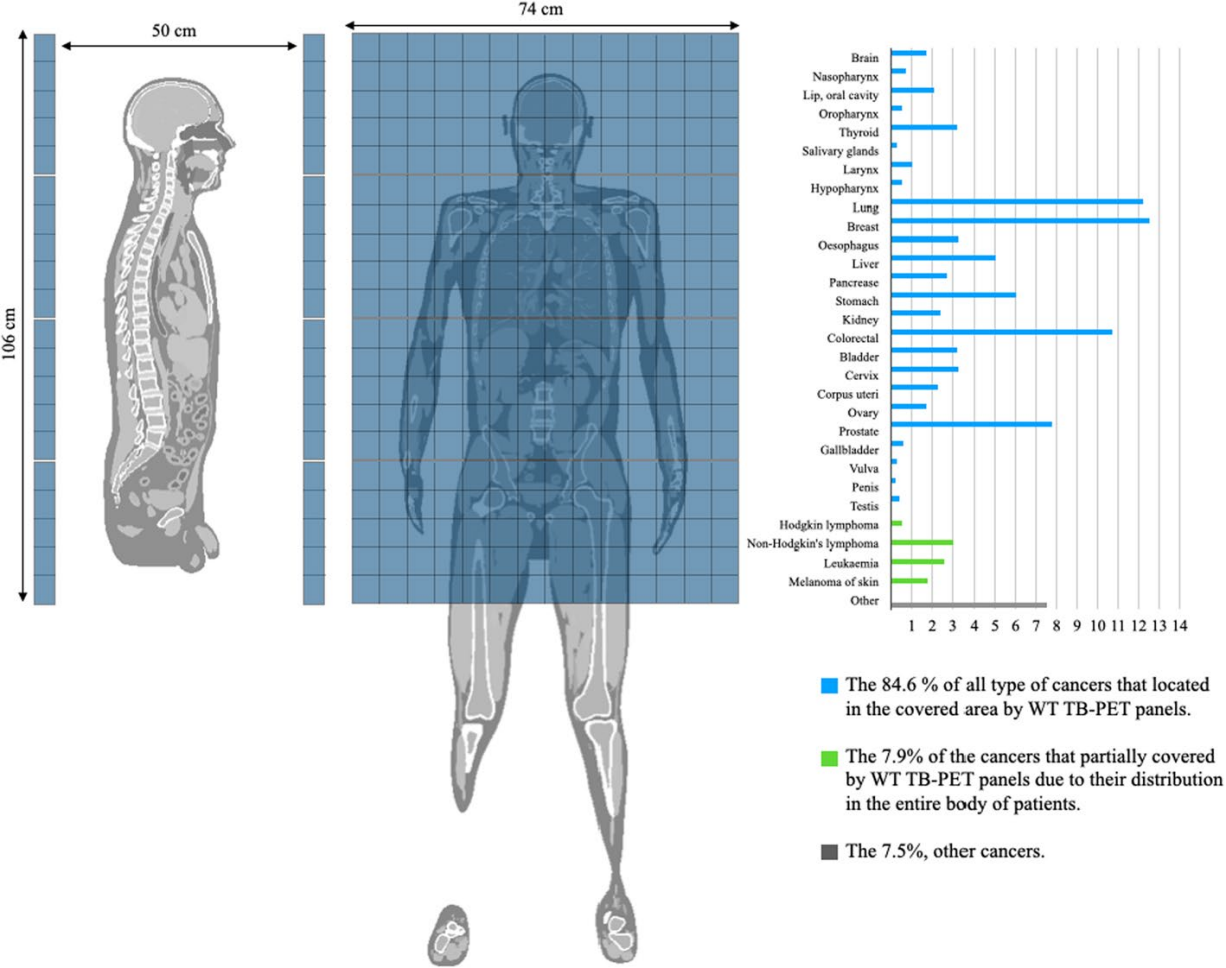
Organ-specific incidence of the most prevalent cancers worldwide. The blue bars indicate cancers that can be fully covered by WT-PET, while the green bars represent cancers that can only be partially covered due to their widespread distribution throughout the patient’s body. The gray bars represent rare cancers in comparison to the other two categories

Figure 1 illustrates the extent of detector coverage provided by the WT-PET panels for organs with a higher cancer incidence based on data from the World Cancer Research Fund in 2020 [25]. In the figure, the blue bars represent the percentage of different cancer types, located within the axial FOV of the WT-PET scanner, accounting for 84.6% of all types of cancers. The green bars represents cancers that are only partially covered by the WT-PET due to their distribution throughout the patient’s entire body, constituting 7.9% of the total. Additionally, the remaining 7.5% (gray bar) represents the cumulative percentage of rare cancers in patients. This implies that the majority of scans can be done in a single patient position, increasing throughput. Based on this data, simultaneous head and torso imaging can provide an optimal trade-off between system cost and detector coverage along the patient’s body. If necessary, it is still possible to image the full patient’s body by moving either the panels or the patient up and down.

The objective of this study is to assess lesion contrast in the WT-PET scanner through the utilization of digital anthropomorphic XCAT phantoms, focusing on three organs with high cancer prevalence: the lung, breast and liver.

## Methods

### Monte Carlo simulation

The study utilized the Geant4 Application for Tomographic Emission (GATE) simulation framework, a Monte Carlo-based software widely recognized for its application in medical physics and nuclear imaging modalities [26, 27]. GATE employs the Geant4 toolkit to accurately simulate the transport of particles through various materials, facilitating precise modeling of radiation interactions and imaging system behaviors. This validated software serves as an advanced and dedicated simulation toolkit, enabling thorough investigation into the characteristics and performance of nuclear imaging modalities. Leveraging its comprehensive and customizable material database, GATE allows for the simulation of both conventional and novel scanners.

The Monte Carlo simulation includes the effects of photon acolinearity, object scatter and attenuation, and crystal scatter. It’s important to note that the optical processes of light emission and transport within the scintillator materials were not incorporated into our simulations. Instead, the detector response to gamma interactions is modelled by incorporating detector energy resolution blurring, detector time resolution blurring, and detector spatial resolution blurring. This was done both for the WT-PET and the LSO LAFOV PET. For the WT-PET, we have used an energy resolution of 15%, coincidence time resolution (CTR) of 327 ps [28], a 2D spatial detector resolution of 1.3 mm FWHM, and a DOI of 2 mm FWHM. For the LSO LAFOV PET, the energy resolution was 11%, the CTR was 228 ps, and the detector spatial resolution was equal to the crystal pixel size (3.2 × 3.2x20 mm^3^). This was done either directly in GATE (energy resolution blurring), or in the pre- processing of the data prior to reconstruction (time/spatial resolution blurring). For a more in-depth overview of the implementation of the scanner parameters, we refer to our previous study [19].

### XCAT anthropomorphic phantoms

XCAT anthropomorphic phantoms are advanced computational models that simulate realistic human anatomy for various medical imaging studies [29–32]. XCAT phantoms provide a valuable tool for assessing imaging system performance, image reconstruction algorithms, and optimizing imaging protocols. These phantoms incorporate anatomical details such as organ shapes, sizes, and tissue densities, allowing for accurate simulations of different patient populations. Additionally, lesions or abnormalities at specific locations within the XCAT phantom can be simulated, enabling the evaluation of lesion contrast and the impact on diagnostic accuracy.

By using XCAT phantoms in this study, we can obtain quantitative measurements and validate the performance of the WT-PET scanner in detecting lesions under controlled and reproducible conditions. In the presented study, the tracer activity for the XCAT phantoms was standardized to 3 MBq/kg [33–35]. The activity has been fixed at a particular distribution, without any further decay modeled during the acquisition itself, since it is only 30 s of data acquisition. Table 2 provides an overview of the characteristics of the XCAT phantoms used in the presented study sorted based on their body mass index (BMI).

**Table 2:** Characteristics of utilized XCAT anthropomorphic phantoms

Phantom ID	Gender	Age	Weight (kg)	Height (cm)	BMI (kg/m^2)
P1	F	27	55.6	172.7	18.64
P2	M	18	62.0	176.0	20.02
P3	F	52	72.0	179.0	22.47
P4	M	31	77.9	185.2	22.71
P5	M	67	89.9	178.5	28.22
P6	M	67	103.3	181.9	31.22
P7	M	64	106.1	175.3	34.53
P8	F	52	86.0	153.0	36.74

### Lesions

**Fig. 2:**
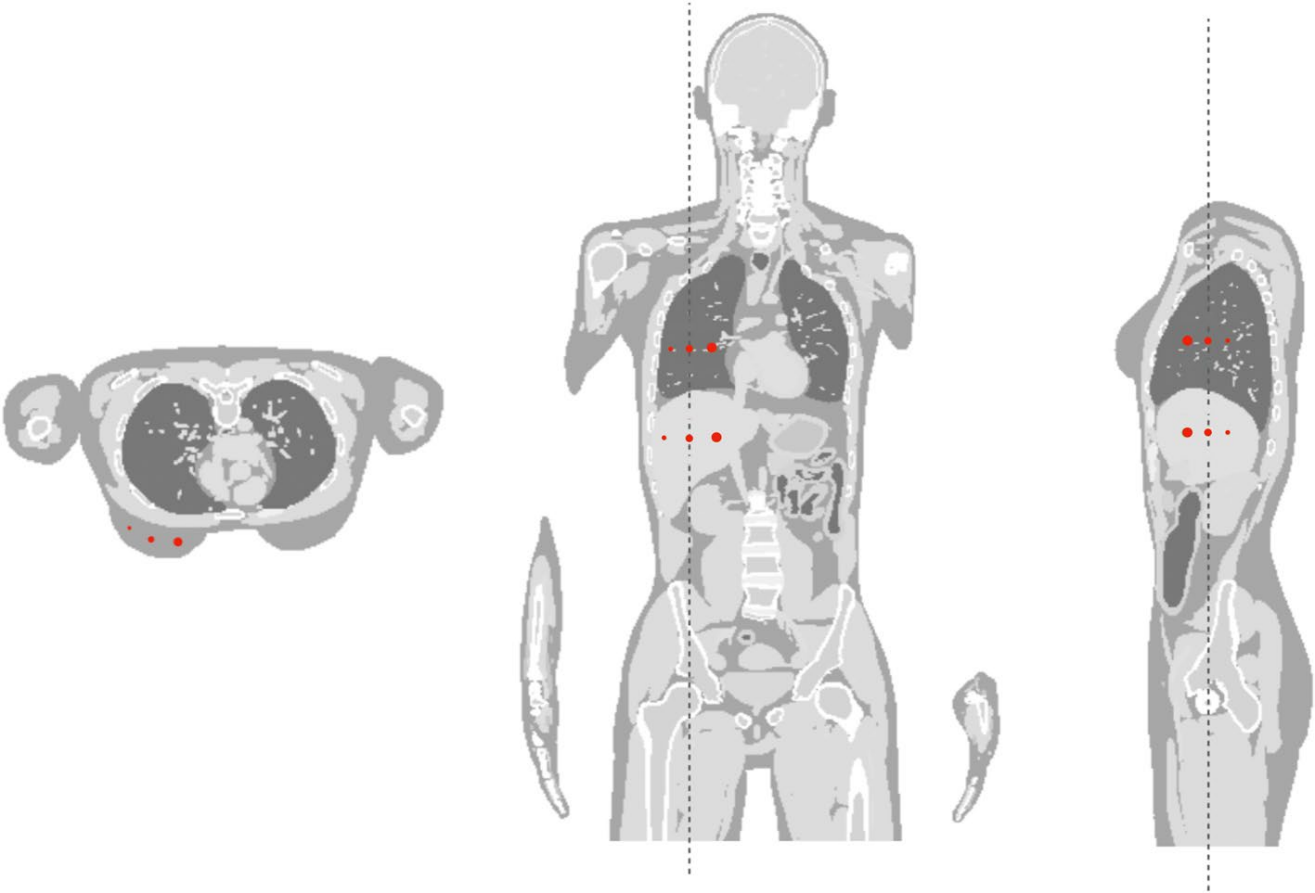
An exemplary illustration of a female XCAT phantom showcasing visible lesions in the lung, liver, and breast. Each organ contains multiple intentionally introduced lesions, with diameters of 5 mm, 7 mm, and 10 mm

The investigation in this study focused on three organs: lung, liver, and breast (female phantoms only). Multiple lesions were intentionally introduced within each organ, with diameters of 5 mm, 7 mm, and 10 mm, and ground-truth tumor-to- background ratios (TBRs) of 2:1, 4:1, and 8:1 [36–38]. Figure 2 shows a female XCAT phantom, displaying the lesion locations in the coronal view of the lung and liver. Four additional lesions were implemented perpendicularly, as seen in the sagittal view. Due to spatial constraints, only three lesions were implemented in the breast: 5 mm, 7 mm, and 10 mm in diameter, with TBR values of 2:1, 4:1, and 8:1 for each, as seen in the transverse view. A separate reconstruction was done for each TBR value.

### Image reconstruction

An iterative list-mode maximum likelihood expectation maximization (MLEM) algorithm with TOF (kernel = 327 ps FWHM [28]) was utilized. For each phantom, 30 s of data acquisition were used for reconstruction. As the investigation did not focus on random/scatter correction, all reconstructions were conducted exclusively using true coincidences, removing randoms and scatters from the list-mode data using the ground truth labels provided by the Monte Carlo simulation. For attenuation correction, the ground-truth attenuation map derived from the XCAT software was employed. All of the reconstructions in the study were performed with a voxel size of 2 mm × 2 mm × 2 mm, using 10 iterations, no subsets and no regularization.

### Quantitive analysis of the lesion contrast

The TBR is a valuable quantitative measure widely employed in medical imaging. It provides a standardized assessment of the relative uptake or concentration of a radiotracer within a tumor compared to the surrounding background tissue. By calculating the (reconstructed) TBR, clinicians and researchers can gauge the level of contrast between the lesion and the normal background, aiding in the accurate identification and characterization of lesions. A higher TBR signifies a more pronounced contrast, indicating a greater likelihood of lesion detection. The TBR is calculated as the mean standardized uptake value (SUVmean) within the volume of interest (VOI) of the tumor, divided by the SUVmean of a VOI in the background tissue (in the same organ). To segment the VOI of the lesions, a threshold of 40% of the maximum standardized uptake value (SUVmax) was applied [39–41]. This thresholding approach involves identifying regions within the image where the SUV values equal or exceed 40% of the maximum SUV value observed in the lesions. By applying this threshold, the VOI boundaries are defined.

When the ground-truth lesion-to-background activity ratio (aratio) is known, the contrast recovery coefficient (CRC) can be calculated from the TBR as:

1 $$CRC=\frac{TBR-1}{{a}_{ratio}-1}$$

Since the CRC is normalized to the ground-truth activity ratio, it allows for easier comparison between lesions of different activity ratios. Therefore, in the presented study, the CRC is being utilized as a quantitative metric to analyze the lesion detectability of the WT-PET scanner.

## Results

**Fig. 3:**
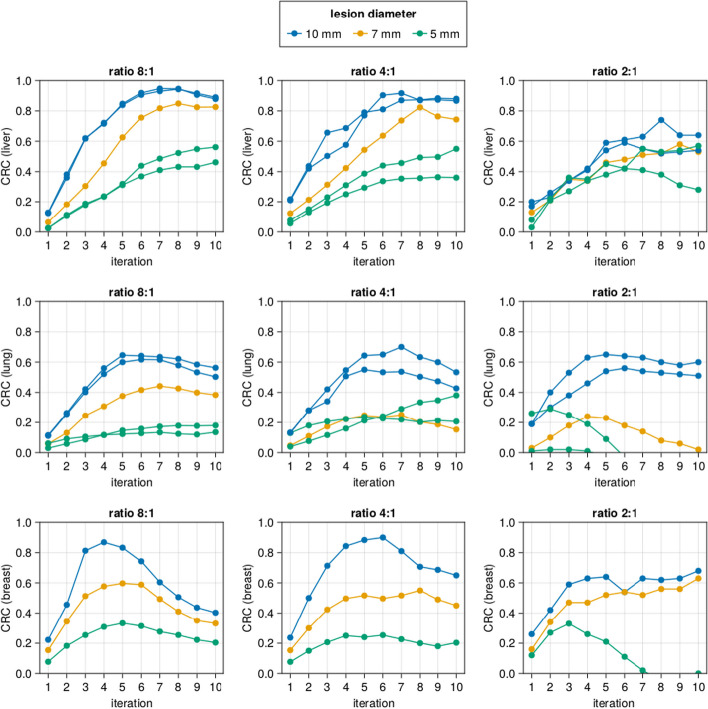
The contrast recovery coefficient (CRC) of different lesions in XCAT phantom P3 (female with BMI of 22.47) in function of the iteration number

In the presented study, lesions were introduced in the liver, lung, and breast of both male and female XCAT phantoms. All the simulations were performed using three different activity concentrations in the lesions (8:1, 4:1, and 2:1). Figure 3 shows the CRC values of the lesions in liver, lung and breast of female XCAT phantom P3 as a function of the iteration number for 8:1, 4:1 and 2:1 lesion to background activity concentration ratios. In a previous study [19], we showed that 10 iterations was sufficient for the convergence of the spatial resolution of point sources in a warm background. Based on Fig. 3, we conclude that further iterations will have little positive effect on the CRC value and will lead to higher noise levels, and fewer than 10 iterations can even be better for specific lesions. Nonetheless, since the optimal iteration number cannot be known beforehand, we will continue with 10 iterations for the rest of this study.

**Fig. 4:**
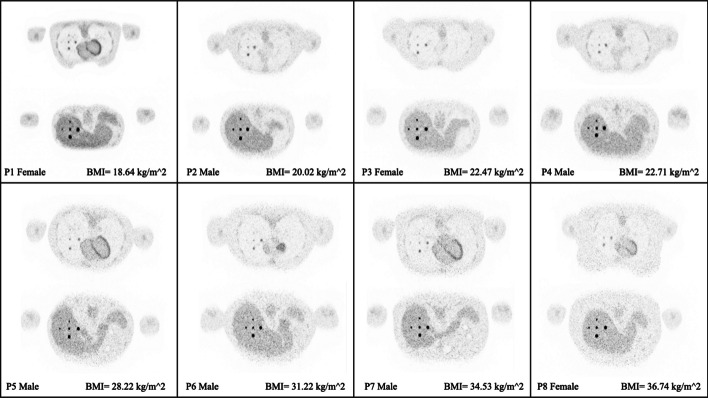
Transverse visualization of the reconstructed images from each of the investigated XCAT phantoms. In each section, the upper image displays the lesions in the lung, while the lower one depicts those located in the liver

**Fig. 5:**
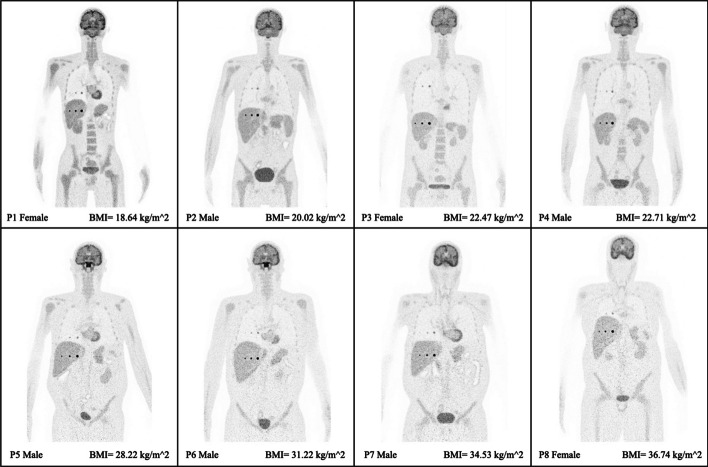
Coronal visualization from the central slice of the XCAT phantoms, where three lesions (10, 7, and 5 mm) in the lungs and liver are visible

**Fig. 6:**
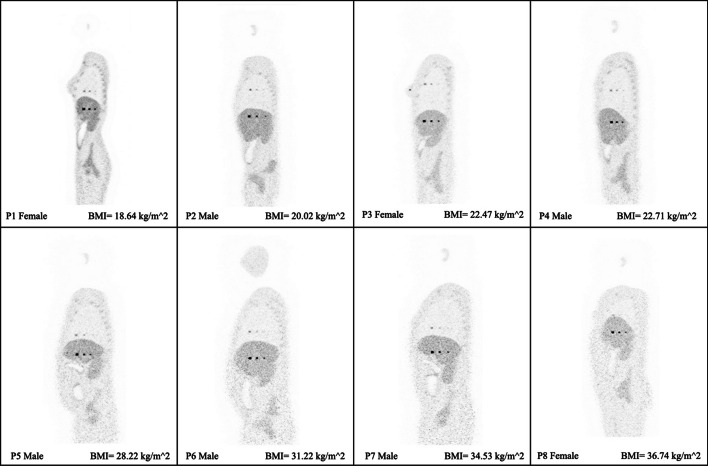
Sagittal illustration of the XCAT phantoms where the three lesions (10, 7 and 5 mm) in the liver and lungs are visible

**Fig. 7:**
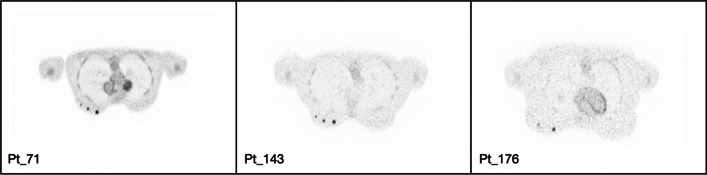
Transverse visualization of the female XCAT phantoms, each featuring three lesions with diameters of 10, 7, and 5 mm in one of the breasts

Figure 4 displays the transverse visualization of the lesions in the lung (upper) and liver (lower) for each of the XCAT phantoms, sorted based on their BMI. To view the reconstructed images from other perspectives, also the coronal (Fig. 5) and sagittal views (Fig. 6) have been illustrated. Since the lesions in the breast are located at different offsets, they can be seen separately in Fig. 7. All visualizations use an 8:1 lesion activity concentration ratio compared to the background tissue.

**Fig. 8:**
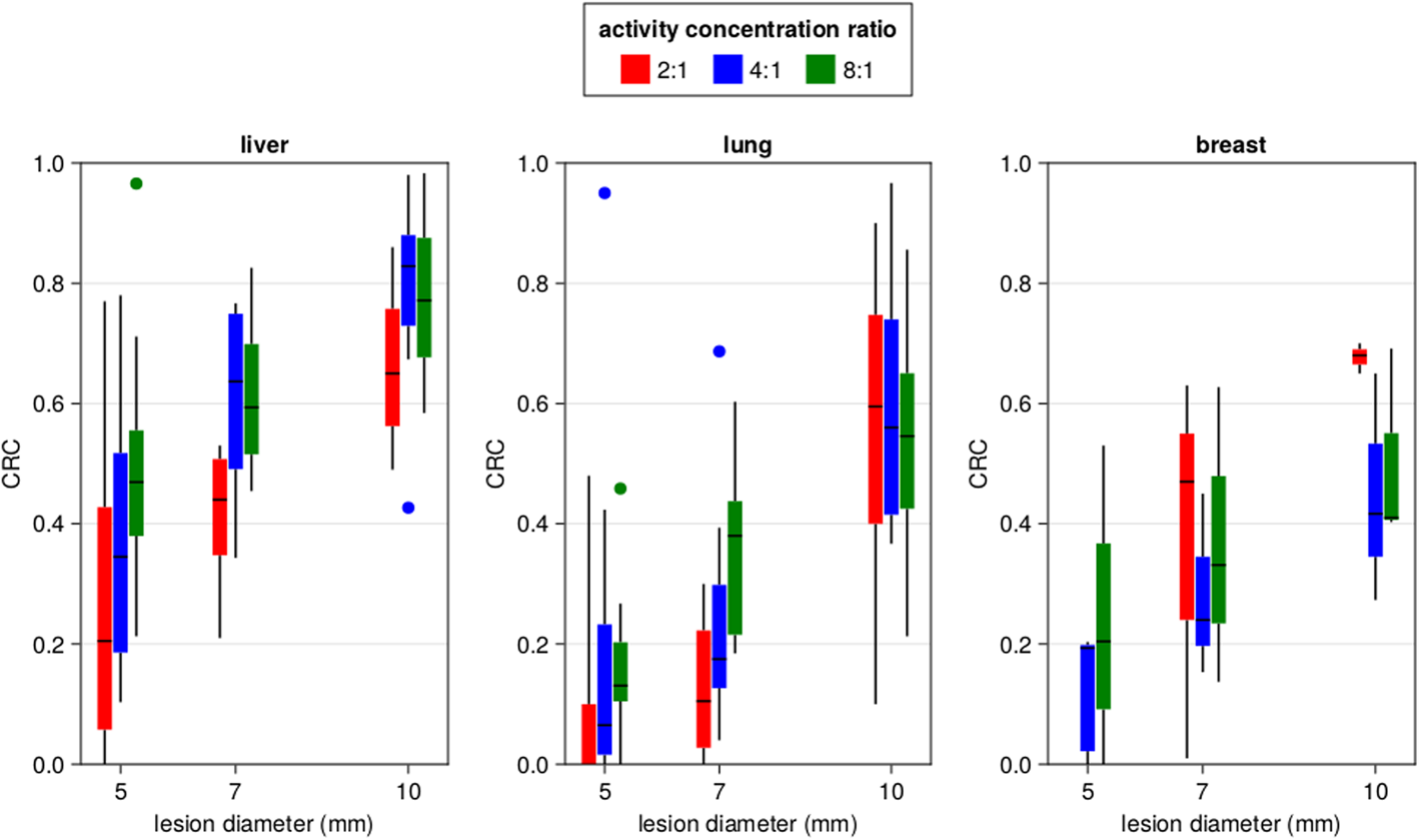
Boxplots showing the contrast recovery coefficient (CRC) in the XCAT phantoms for different lesion diameters and activity concentration ratios in the liver, lung and breast

Figure 8 shows a summary of the calculated CRC values for all lesion locations, sizes and activity concentration ratios over the various XCAT phantoms. Overall, the CRC values for lesions in the liver are the largest, and those in the lung the lowest. This is likely due to the higher uptake values in the liver and lower uptake values in the lung, leading to different coincidence rates (and therefore noise statistics) for the same TBR value.

**Fig. 9:**
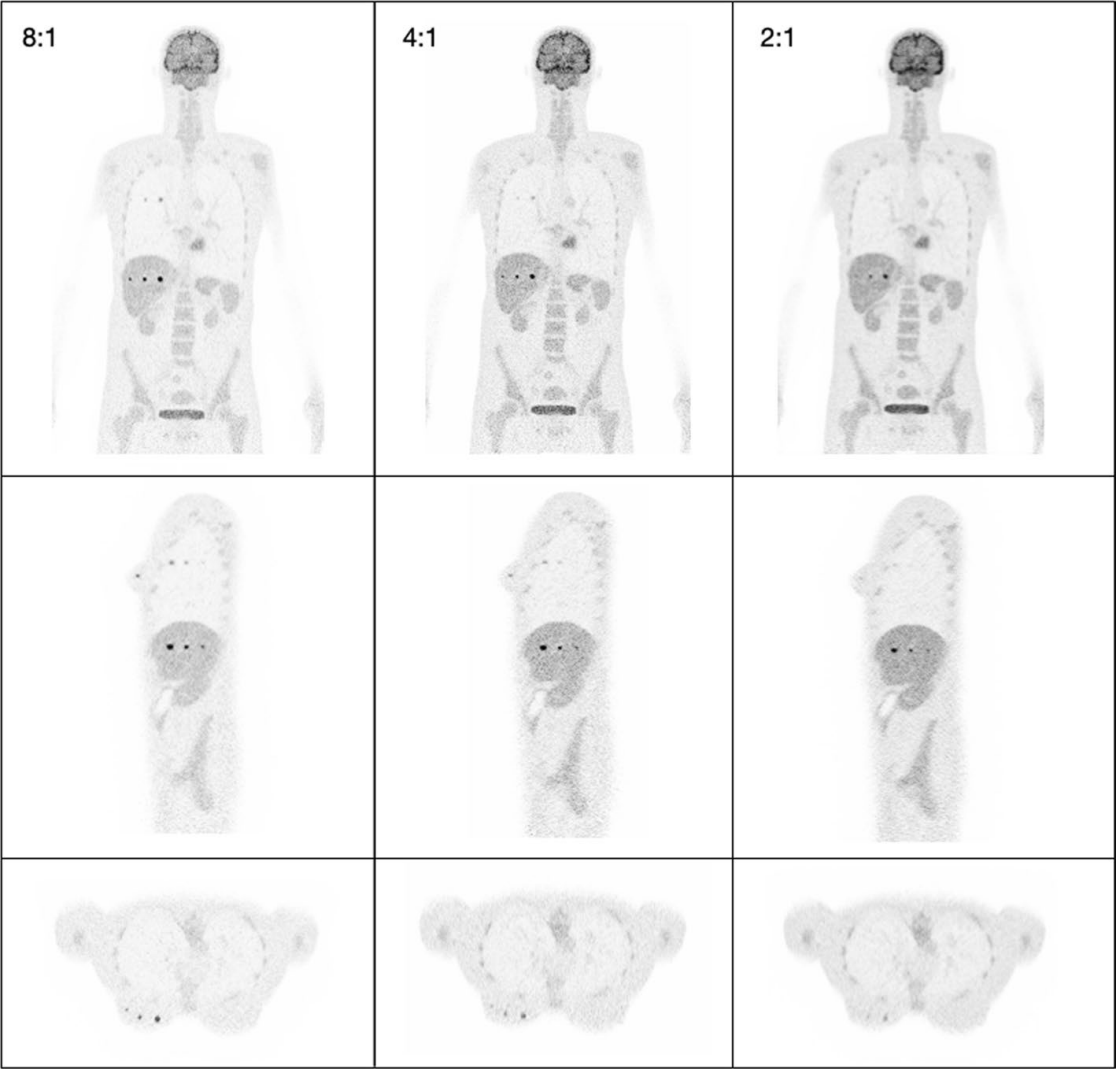
Reconstructed images (10th iteration) of a female XCAT phantom (P3) displaying various lesion activity concentrations (8:1, 4:1, and 2:1) in coronal (upper), sagittal (middle), and transverse (lower) slices

To visually demonstrate the effect of varying lesion activity concentrations relative to the background tissue’s activity, we selected XCAT phantom P3 (female with BMI of 22.47) as a representative example, showed in Fig. 9.

## Discussion

The primary objective of our study revolved around evaluating the lesion contrast capabilities in the WT-PET scanner for standard dose and with very short scan times of 30 s, using XCAT anthropomorphic phantoms. The study shows a correlation between the CRC value and the diameter of the lesion, with higher CRC values obtained for larger lesions. It is particularly noteworthy that the liver manifested higher average CRC values when compared with the lung and breast. This can be attributed to the liver’s higher uptake value compared to the lungs and breasts. Such an understanding can guide clinicians in weighing the confidence they place on PET results, depending on the organ under examination.

Our findings suggest a pivotal challenge in the detection of smaller lesions (5 mm and 7 mm) in the lungs, especially at lower activity concentrations. Similarly, the 5 mm lesions in the breast are difficult to detect, although there the CRC does increase rapidly for increasing activity ratio. Given that early-stage lung cancers might be characterized by such small lesion sizes, the findings underscore a crucial limitation that must be considered when leveraging the WT-PET for lung and/or breast cancer detection. Strategies might need to be developed to enhance detectability in these cases. For example, advanced regularization schemes could be used to allow much higher iteration numbers while suppressing noise characteristics, thereby improving lesion detectability. Regularization was not included in the current study.

While our study has shown the capabilities of the WT-PET scanner, understanding how it fares against conventional PET configurations is crucial. This helps in placing our findings in the broader context of nuclear imaging, providing clinical practitioners a more comprehensive understanding of where the WT-PET scanner stands in the pantheon of available imaging modalities. Therefore, we perform a benchmarking exercise comparing our results to a more conventional LAFOV PET, based on pixelated LSO detector technology, possessing attributes analogous to the Quadra. It should be noted that these reconstructions are not necessarily representative of what would be obtained on the actual Quadra PET scanner, given the differences in event processing, image reconstruction algorithm, regularization, etc. Instead, these reconstructions are meant to offer a comparison of lesion contrast between the WT-PET and a conventional cylindrical PET with similar axial FOV (henceforth referred to as LSO LAFOV PET), changing only the geometry and detector parameters.

**Fig. 10:**
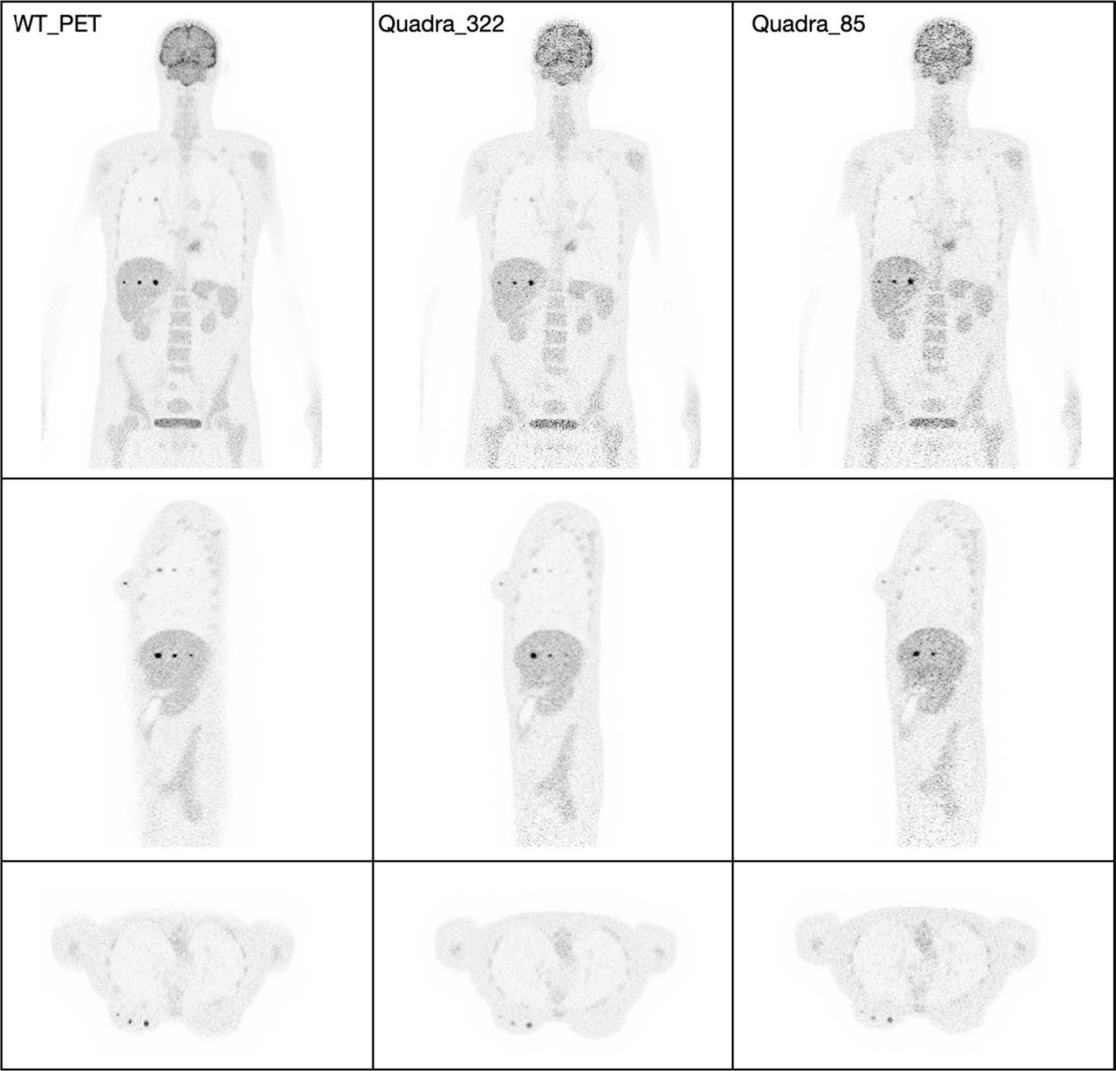
Illustration of the reconstructed image (10th iteration) of the P3 female XCAT phantom by WT-PET, LSO LAFOV PET 322 MRD, and 85 MRD with 8:1 of lesion activity concentration ratio to background

**Fig. 11:**
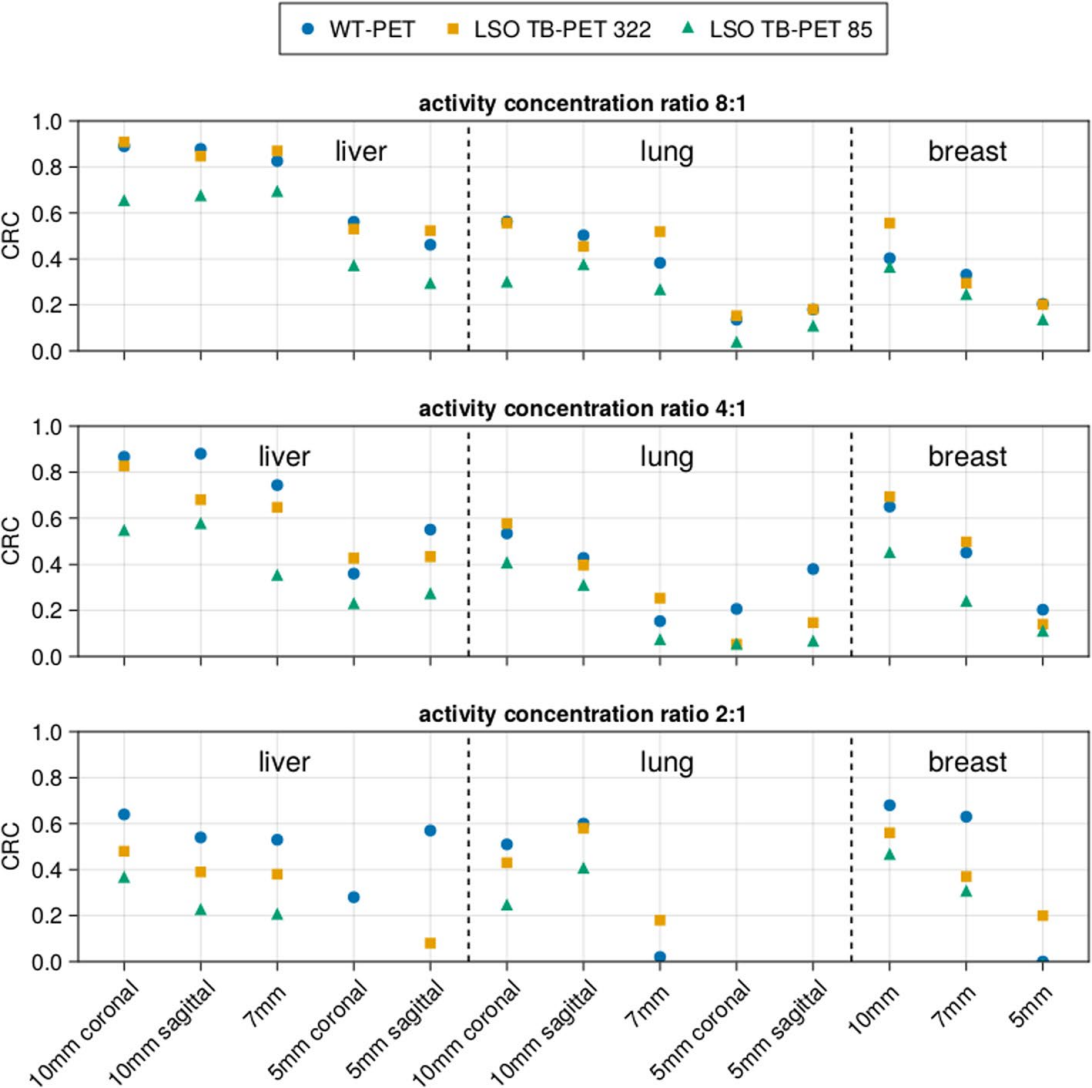
Contrast recovery coefficient (CRC) of lesions in XCAT phantom P3 (female of BMI 22.47), comparing the WT-PET with a conventional LSO LAFOV PET scanner with the same AFOV, with and without a maximum ring difference (MRD) cut

**Fig. 12:**
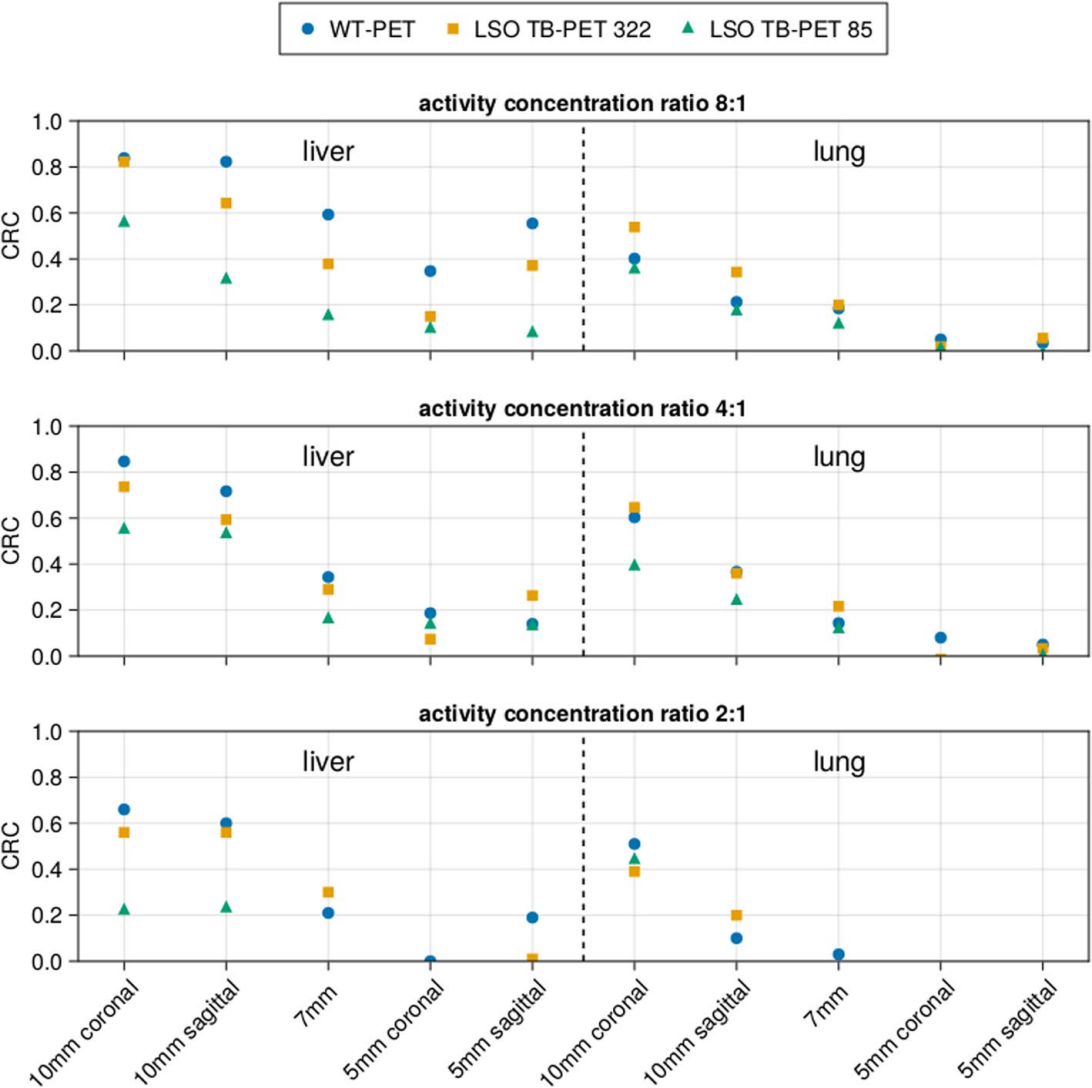
Contrast recovery coefficient (CRC) of lesions in a XCAT phantom P4 (male of BMI 22.71), comparing the WT-PET with a conventional LSO LAFOV PET scanner with the same AFOV, with and without a maximum ring difference (MRD) cut

Figure 10 is an exemplary visualization of the reconstructed image by performing the simulation from a female XCAT phantom in the WT-PET and the LSO LAFOV PET with no maximum ring difference cut (322 MRD) and an 85 MRD cut at the 10th iteration. To have a better comparison, the TBR values have been calculated for each activity concentration as shown in Fig. 11. A male XCAT phantom (P4) has additionally been reconstructed (but not visualized) to have an example from both genders, with TBR values shown in Fig. 12.

The results in Figs. 11 and 12 show that the CRC values of the LSO LAFOV PET 322 MRD are similar to those of the WT-PET. However, the WT-PET has a higher recovered contrast than the LSO LAFOV PET with 85 MRD. For example, in case of the WT-PET all liver lesions and some lung lesions at a lower activity concentration ratio (2:1) were visually distinguished on the image, while for the LSO LAFOV PET with 85 MRD only the 10 mm diameter lesions in these organs were apparent. The LSO LAFOV PET with 322 MRD performs better than 85 MRD, as expected because the 85 MRD cut reduces the sensitivity of the LSO LAFOV PET by almost half.

The WT-PET demonstrated an improvement in the average CRC values. Specifically, the CRC was increased on average by 0.10 and 0.29 in the liver, 0.01 and 0.10 in the lung, and 0.00 and 0.14 in the breast, compared to the LSO LAFOV PET 322 MRD and 85 MRD, respectively.

Therefore, one of the main advantages of the WT-PET is the ability to maintain good lesion contrast despite using less scintillation material and fewer detectors (around 50% less for the same AFOV). This is achieved through a combination of the high-resolution DOI capable detectors, and the proximity of the detectors to the patient, increasing sensitivity.

**Fig. 13:**
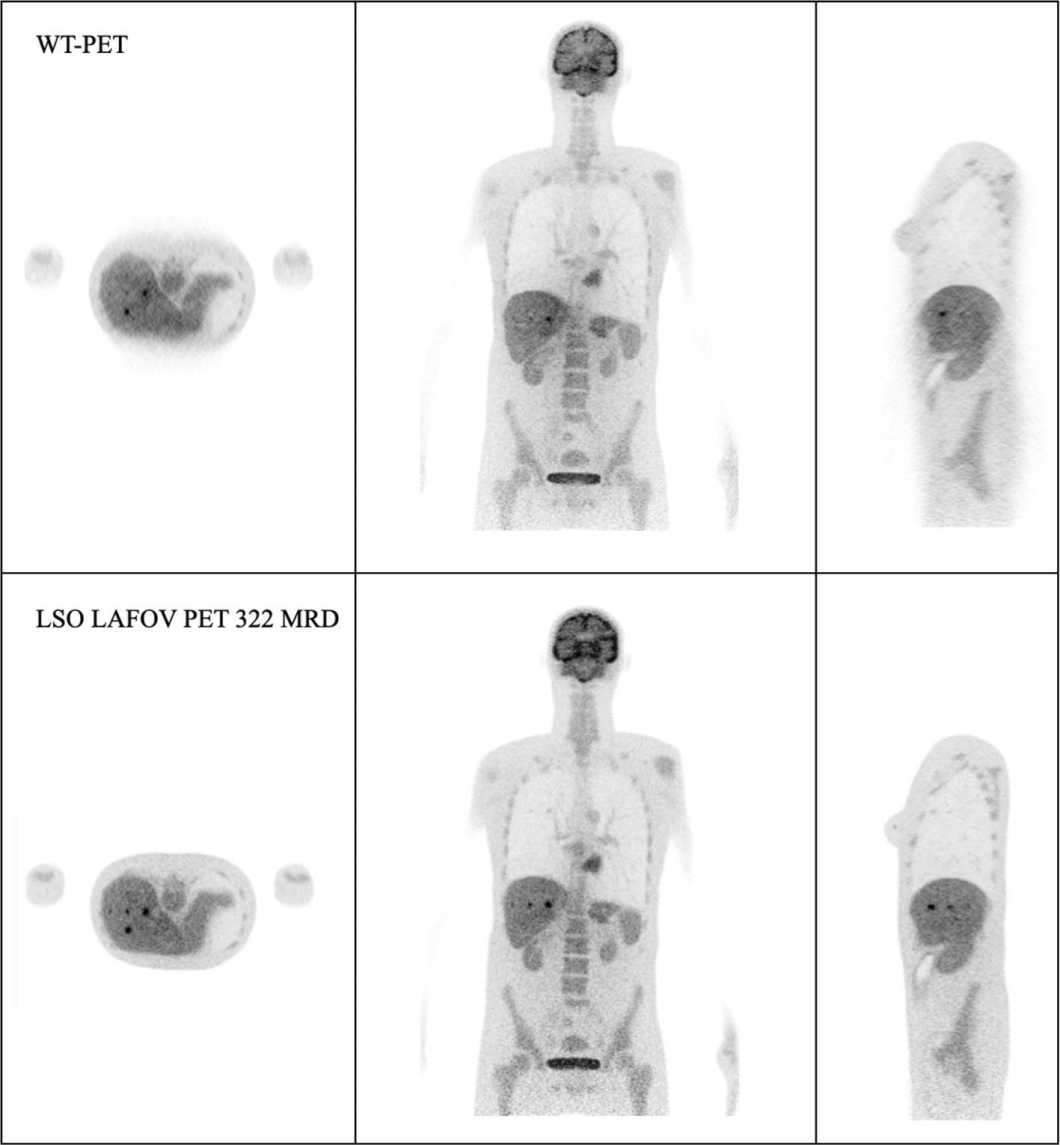
Illustration of the reconstructed image (10th iteration) with all type of the coincidences, of the P3 female XCAT phantom by WT-PET, LSO LAFOV PET 322 MRD, with 8:1 of lesion activity concentration ratio to background

**Fig. 14:**
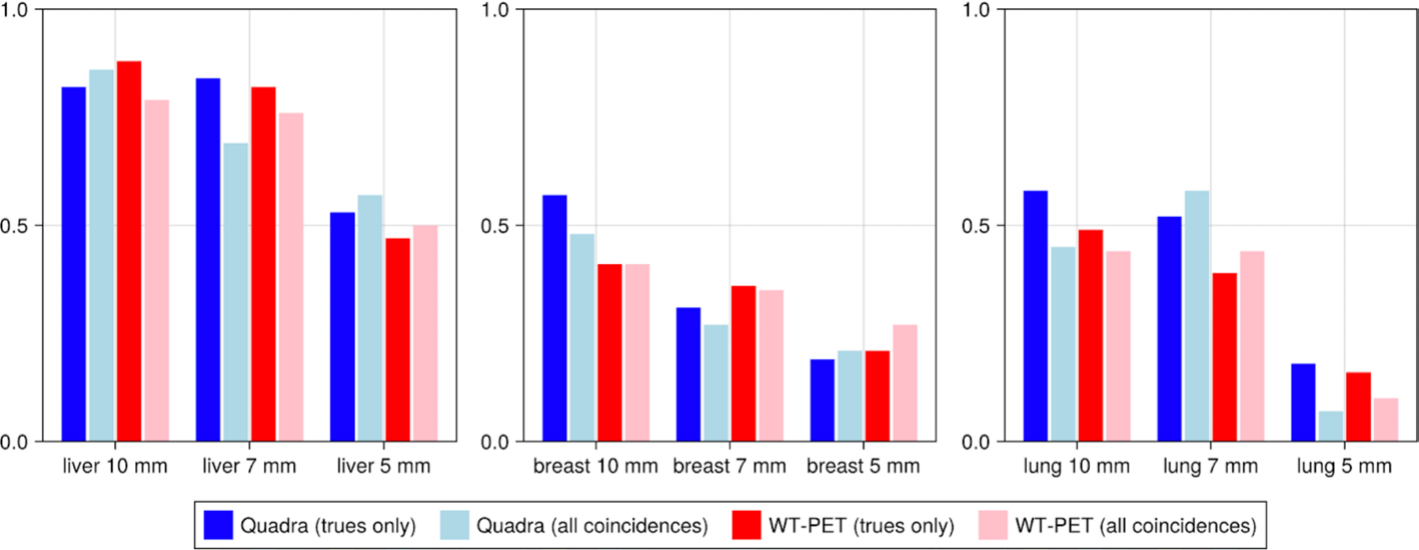
Contrast recovery coefficient (CRC) of lesions in an XCAT phantom (P3), comparing reconstructions utilizing true and all coincidences between the WT-PET and a conventional LSO LAFOV PET scanner

To assess the potential impact of randoms and scatters, the P3 phantom was reconstructed using all coincidences (true, random and scattered), but without random and scatter correction. This was done for both the WT-PET and the LSO LAFOV, and the reconstructions are shown in Fig. 13. Subsequently, the lesion CRC values were calculated, and compared to the true coincidence only reconstructions, see Fig. 14.

We do not observe significant differences in CRC between reconstructions using true coincidences only and reconstructions utilizing all coincidences. However, for future research, we plan to implement random and scatter corrections in the WT-PET system.

It is important to note that patient motion can also affect image quality. In the presented study the patient’s motion has not been modeled. While increased motion is expected for standing patients, the short scan duration (30 s) reduces its effects. We have previously explored the range of patient motion through the participation of volunteers, where the average movement of various body parts, including the head, shoulders, chest and abdomen was tracked. The average motion was in the range of 3–10 mm FWHM, indeed above the spatial resolution of 2 mm [42, 43]. This was however done without any patient support for the back and/or neck, which we believe would further reduce the range of motion. We are currently in the process of designing and evaluating a patient support system to bring patient motion more in line with the system’s spatial resolution.

The design aims to set itself apart from other LAFOV PET scanners by providing: i) higher patient throughput, achieved by the high sensitivity and no more need for patient positioning on a bed, ii) high spatial resolution, achieved by the high- resolution DOI capable monolithic detectors, and iii) a cost reduction compared to similar axial FOV scanners, achieved by the reduced detector area required for the flat-panel geometry and the use of monolithic (BGO) detectors.

## Conclusion

The presented results affirm the WT-PET scanner’s ability to identify the small lesions with low activity concentration in the liver as a vital organ. The detailed inspection of the results presented in this study distinctly illustrates the capabilities of the WT-PET scanner relative to more conventional pixelated LSO LAFOV PET scanners. The WT PET necessitates around 50% fewer scintillation crystals and SiPM compared to customary PET scanners sharing a similar AFOV (106 cm). This reduction does not compromise its efficacy in lesion detection, a critical functionality in PET scanners, thus presenting a promising horizon in balancing performance and affordability in PET scanner development.

## Data Availability

The data used and/or analysed during the current study are available from the corresponding author upon request.

## References

[1] Ilcheva M, Nikolova P, Hadzhiyska V, Mladenov K (2022). Impact of FDG PET/CT on detection of synchronous and metachronous malignancies and clinical management in patients with multiple primary cancers. Neoplasma.

[2] Vandenberghe S, Moskal P, Karp J (2020). State of the art in total body PET. EJNMMI Phys.

[3] Dadgar M, Parzych S, Tayefi Ardebili F. A simulation study to estimate optimum LOR angular acceptance for the image reconstruction with the Total-Body J-PET. In Annual conference on medical image understanding and analysis 2021 (pp. 189-200). Cham: Springer International Publishing. 10.1007/978-3-030-80432-915

[4] Moskal P, Stepien E (2020). Prospects and clinical perspectives of total-body pet imaging using plastic scintillators. PET Clin.

[5] Spencer B, Berg E, Schmall J, Omidvari N (2021). Performance evaluation of the uEXPLORER total-body PET/CT scanner based on NEMA NU 2–2018 with additional tests to characterize PET scanners with a long axial field of view. J Nucl Med.

[6] Karp J, Viswanath V, Geagan M, Muehllehner G, Pantel A (2020). Pennpet explorer: design and preliminary performance of a whole-body imager. J Nucl Med.

[7] Pantel A, Viswanath V, Daube-Witherspoon M, Dubroff J (2020). Pennpet explorer: human imaging on a whole-body imager. J Nucl Med.

[8] Alberts I, Hünermund JN, Prenosil G, Mingels C, Bohn KP, Viscione M, Sari H, Vollnberg B, Shi K, Afshar-Oromieh A, Rominger A (2021). Clinical performance of long axial field of view PET/CT: a head-to-head intra-individual comparison of the biograph vision Quadra with the biograph vision PET/CT. Eur J Nucl Med Mol Imaging.

[9] Prenosil GA, Sari H, Fürstner M, Afshar-Oromieh A, Shi K, Rominger A, Hentschel M (2022). Performance characteristics of the biograph vision Quadra PET/CT system with a long axial field of view using the NEMA NU 2–2018 standard. J Nucl Med.

[10] Cherry S, Jones T, Karp J, Qi J (2018). Total-body pet: maximizing sensitivity to create new opportunities for clinical research and patient care. J Nucl Med.

[11] Dadgar M, Parzych S, Baran J (2023). Comparative studies of the sensitivities of sparse and full geometries of total-body pet scanners built from crystals and plastic scintillators. EJNMMI Phys.

[12] Vandenberghe S, Muller F, Withofs N, Dadgar M (2023). Walk-through flat panel total-body PET: a patient-centered design for high throughput imaging at lower cost using DOI-capable high-resolution monolithic detectors. Eur J Nucl Med Mol Imaging.

[13] Vandenberghe S, Karakatsanis NA, Akl MA, Maebe J, Surti S, Dierckx RA, Pryma DA, Nehmeh SA, Bouhali O, Karp JS (2023). The potential of a medium-cost long axial FOV PET system for nuclear medicine departments. Eur J Nucl Med Mol Imaging.

[14] Abi-Akl M, Dadgar M, Toufique Y (2023). Monte Carlo simulation of the system performance of a long axial field-of-view pet based on monolithic lyso detectors. EJNMMI Phys.

[15] Dadgar M, Kowalski P (2020). Gate simulation study of the 24-module j-pet scanner: data analysis and image reconstruction. Acta Phys Pol B.

[16] Borys D, Baran J, Brzezinski K (2022). Protheramon—a gate simulation framework for proton therapy range monitoring using pet imaging. Phys Med Biol.

[17] Dadgar M, Parzych S, Tayefi Ardebili F (2023). Investigation of novel preclinical total body pet designed with J-PET technology: a simulation study. IEEE Trans Radiat Plasma Med Sci.

[18] Dadgar M, Parzych S, Tayefi Ardebili F, Moskal P, Vandenberghe S (2022). Introduction of the DOI capable Total-Body J-PET, a simulation study. J Nucl Med..

[19] Dadgar M, Maebe J, Abi Akl M, Vervenne B, Vandenberghe S (2023). A simulation study of the system characteristics for a long axial FOV PET design based on monolithic BGO flat panels compared with a pixelated LSO cylindrical design. EJNMMI Phys.

[20] Vandenberghe S, Abi Akl M, Withofs N, Muller FM, Maebe J, Dadgar M, et al. Efficient patient throughput and detector usage in low cost efficient monolithic high resolution walk-through flat panel total body PET. In: Total-Body PET 2022, Abstracts. 2022. pp. 28–29

[21] Maebe J, Vandenberghe S (2023). Effect of detector geometry and surface finish on cerenkov based time estimation in monolithic BGO detectors. Phys Med Biol.

[22] Maebe J, Vandenberghe S (2022). Simulation study on 3D convolutional neural networks for time-of-flight prediction in monolithic pet detectors using digitized waveforms. Phys Med Biol.

[23] Muller F, Vanhove C, Vandeghinste B, Vandenberghe S (2022). Performance evaluation of a micro-ct system for laboratory animal imaging with iterative reconstruction capabilities. Med Phys.

[24] Stockhoff M, Van Holen R, Vandenberghe S (2019). Optical simulation study on the spatial resolution of a thick monolithic pet detector. Phys Med Biol.

[25] Cancer data: World cancer research fund international, W. WCRF International https://www.wcrf.org/cancer-trends/worldwide-cancer-data/ (2022).

[26] Sarrut D, Bala M, Bardies M (2021). Advanced monte carlo simulations of emission tomography imaging systems with gate. Phys Med Biol.

[27] Moskal P, Kowalski P, Shopa R, Raczynski L (2021). Simulating nema characteristics of the modular total-body J-PET scanner-an economic total-body pet from plastic scintillators. Phys Med Biol.

[28] Carra P, Giuseppina Bisogni M, Ciarrocchi E, Morrocchi M, Sportelli G, Rosso V, Belcari N (2022). A neural network-based algorithm for simultaneous event positioning and timestamping in monolithic scintillators. Phys Med Biol.

[29] Segars W, Sturgeon G, Mendonca S, Grimes J, Tsui B (2010). 4D XCAT phantom for multimodality imaging research. Med Phys.

[30] Segars W, Veress A, Sturgeon G, Samei E (2019). Incorporation of the living heart model into the 4D XCAT phantom for cardiac imaging research. IEEE Trans Radiat Plasma Med Sci.

[31] Fedrigo R, Segars W, Martineau P, Gowdy C (2022). Development of scalable lymphatic system in the 4D XCAT phantom: application to quantitative evaluation of lymphoma pet segmentations. Med Phys.

[32] Segars W, Bond J, Frush J, Hon S (2013). Population of anatomically variable 4D XCAT adult phantoms for imaging research and optimization. Med Phys.

[33] Van Sluis J, Boellaard R, Dierckx R, Stormezand G (2020). Image quality and activity optimization in oncologic 18f-fdg pet using the digital biograph vision PET/CT system. J Nucl Med.

[34] Gruber J, Decristoforo C, Uprimny P, Schoenberg S (2022). Imaging properties and tumor targeting of 68ga-neobomb1, a gastrin-releasing peptide receptor antagonist, in gist patients. Biomedicines.

[35] Nievelstein RA, Quarles van Ufford HM, Kwee TC, Bierings MB, Ludwig I, Beek FJ, de Klerk JM, Mali WP, de Bruin PW, Geleijns J (2012). Radiation exposure and mortality risk from CT and PET imaging of patients with malignant lymphoma. Eur Radiol.

[36] Cheng X, Yang D, Zhong Y, Shao Y (2022). Real-time marker-less tumor tracking with TOF PET: in silicofeasibility study. Phys Med Biol.

[37] Adu-Poku O (2022). Image quality assessment using NEMA standards for lu-177 radionuclide. IJMPCERO.

[38] Saaidi R (2024). Monte Carlo simulation of two siemens biograph PET/CT system using gate: image quality performance. Radiat Phys Chem.

[39] Lawrence E, Kieler M, Cooley G, Wells S, Cho S (2023). Assessment of 18f-dcfpyl PSMA PET/CT and PET/MR quantitative parameters for reference standard organs: inter-reader, inter-modality, and inter-patient variability. PLoS ONE.

[40] Gu J, Khong P, Wang S, Chan Q, Law W, Zhang J (2011). Quantitative assessment of diffusion-weighted MR imaging in patients with primary rectal cancer: correlation with FDG-PET/CT. Mol Imaging Biol.

[41] Nestle U, Kremp S, Schaefer-Schuler A, Sebastian-Welsch C, Hellwig D, Rübe C, Kirsch CM (2005). Comparison of different methods for delineation of 18F-FDG PET–positive tissue for target volume definition in radiotherapy of patients with non–small cell lung cancer. J Nucl Med.

[42] Maebe J, Muller FM, Withofs N, Abi Akl M, Dadgar M, Vanhove C, Vandenberghe S. Walk-through flat panel total body PET: a novel scanner design for efficient patient throughput and detector usage. In 20th national day on biomedical engineering 2022.

[43] Muller FM, Maebe J, Dadgar M, Withofs N, Vanhove C, Vandenberghe S. Rigid body motion analysis in walk-through total body PET scanner based on real-time motion tracking with cameras: comparative study between free-breathing and breath-hold. In Total-Body PET 2022 2022.

